# ANOMALY: a Snakemake pipeline for identifying NuMTs from long-read sequencing data

**DOI:** 10.1093/nargab/lqag014

**Published:** 2026-02-04

**Authors:** Nirmal S Mahar, Rachit Singh, Ishaan Gupta, Shweta Ramdas

**Affiliations:** Department of Biochemical Engineering and Biotechnology, Indian Institute of Technology Delhi, New Delhi 110016, India; Centre for Brain Research, Indian Institute of Science, Bangalore 560012, India; Department of Biochemical Engineering and Biotechnology, Indian Institute of Technology Delhi, New Delhi 110016, India; Centre for Brain Research, Indian Institute of Science, Bangalore 560012, India

## Abstract

Nuclear mitochondrial DNA segments (NuMTs) can contribute to cancer development and disease progression by disrupting protein-coding genes. Furthermore, their presence confounds mitochondrial variant detection, underscoring the critical need for robust NuMT detection. Current methods to call NuMTs rely on short-read sequencing data but struggle to resolve complex NuMTs. These limitations can be overcome by employing long-read sequencing data. However, no such workflow exists to capture NuMTs from long-read sequencing data. Here, we introduce ANOMALY, a novel, easy-to-use workflow for detecting NuMTs from long-read sequencing data. The pipeline takes raw sequencing or aligned data and calls and visualises sample NuMTs. On 50 simulated datasets, the pipeline demonstrated high accuracy, with a precision of 1.000, a recall of 0.989, and an F1-score of 0.994. The pipeline underscores the limitations of short-read data in resolving and capturing complex NuMTs while demonstrating that long-read data enables their accurate identification. The Snakemake pipeline employs Python, Bash and R and is published under an open-source GNU GPL v3 license. Detailed information on setting up and running the pipeline, along with the source code, is available at https://github.com/Nirmal2310/ANOMALY.

## Background

Nuclear mitochondrial DNA segments (NuMTs) represent segments of mitochondria-derived DNA in the nuclear genome. These insertions are estimated to occur at a rate of ∼ 5 × 10^−6^ per base pair per generation in humans [1]. A recent study utilising whole genome sequences from 66,083 samples has determined that each individual carries 4.7 NuMT events on average [2]. These NuMTs have been implicated in genome function [3] and disease [4–7], and used in phylogenetic inference [8]. Accurate NuMT detection is also critical for clinical and population genetics, as undetected NuMTs can be misinterpreted as low-level heteroplasmies, leading to false-positive mitochondrial variants [3, 9].

Existing tools for calling NuMTs from whole genome sequencing data, including DINUMT [10] and the approach proposed by Wei *et al.* [11], are based on short paired-end reads only. With the increasing adoption and availability of long-read sequencing technologies in both small and population-scale genomic studies, there is a need for a tool that can characterise NuMTs from these datasets. Long reads offer higher accuracy in calling structural variants [12], including long insertions like NuMTs; several tools have been built specifically for this purpose and have led to the discovery of novel structural variations in human populations. While tools like PALMER [13] can be repurposed for capturing NuMT, it is not optimised to capture broad ranges of NuMTs, such as concatenated NuMTs. Hence, no such tool exists specifically optimised for discovering novel NuMTs from long-read sequencing data.

We present a novel Snakemake-based pipeline called ANOMALY (**A**nalysis of **N**uclear inserts **O**f **M**itochondri**A** using **L**ong-read sequencing in **Y**our data) for NuMT calling from long-read whole-genome sequencing data. ANOMALY is a pipeline based on existing state-of-the-art open-source methods. Implemented in Snakemake [14], the tool (with its necessary dependencies) is easy to install and quick to run (median execution time of 480.6 seconds using 16 computational threads, Table 1). In simulated data based on the T2T-CHM13 genome [15], ANOMALY identified 540 out of 546 simulated NuMTs with a false negative rate of 0.011 (Supplementary Table 1).

**Table 1.** Performance comparison of NuMT detection tools on simulated datasets

Method	DINUMT	Wei *et al*	PALMER	ANOMALY
**Sequencing Data**	Short-Read	Short-Read	Long-Read	Long-Read
**True Positive (TP)**	153	121	167	168
**False Positive (FP)**	0	0	0	0
**False Negative (FN)**	17	49	3	2
**Precision**	1	1	1	1
**Recall**	0.9	0.71	0.982	0.988
**F1 Score**	0.95	0.83	0.991	0.994
**Median Execution Time (Seconds)**	24.62	4815.21	4081.85	480.59
**Peak Memory Usage (GB)**	0.02	3.81	38.58	3.28
**Number of Threads**	1	1	1	16

## Materials and methods

### Overview

**Figure 1.**
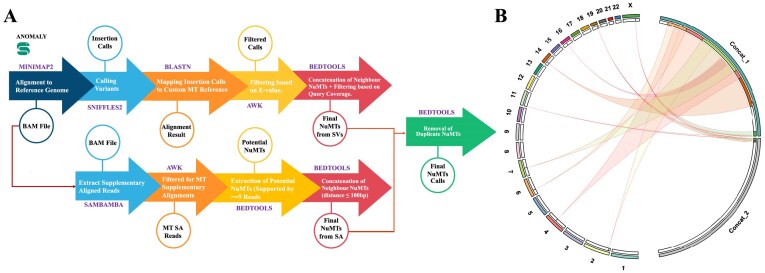
**(A)** ANOMALY workflow. The workflow is implemented as a Snakemake pipeline designed to detect NuMTs. It accepts raw sequencing data in FASTQ format or pre-aligned data in BAM format as input. The pipeline produces a TSV file containing NuMT calls and visual representations as a Circos plot, saved in PNG and SVG formats. The open-source tools involved in each step are also listed in the schematic representation. The steps are described along the arrows connecting the workflow components. Outputs of each step are indicated within circles. Custom MT Reference in the figure represents the concatenated mitochondrial genome. The concatenated mitochondrial genome is employed to capture NuMTs containing the D-loop of the mitochondrial genome. (**B)** The Circos plot illustrates the integration of Nuclear Mitochondrial DNA Sequences (NuMTs) across various chromosomes in the nuclear genome. The outermost circular segments represent individual chromosomes (1–22 and X) and concatenated mitochondrial genome segments (Concat_1 and Concat_2). The curved links in the inner region indicate the insertion events of NuMTs from the mitochondrial genome into nuclear chromosomes. To visualise the NuMTs containing the control region of the mitochondrial genome, we have utilised the concatenated genome of Mitochondria (shown as Concat_1 and Concat_2). Hence, all the NuMTs containing control regions will be split into two links, one coming from the end of the first mitochondrial segment (Concat_1) and the second coming from the start of the second mitochondrial segment (Concat_2).

ANOMALY is a UNIX-based Snakemake pipeline that takes as input raw sequencing data, or whole-genome sequenced aligned data (Fig. 1A)—using the existing structural variant (SV) caller Sniffles2 [16], the pipeline first calls all structural variants (SV) in the sample (step 1). The sequence of each identified insertion is then aligned to the concatenated reference mitochondrial genome FASTA using Nuclear BLAST [17]. The concatenated reference mitochondrial FASTA file used for this local alignment is generated using SeqKit [18] to account for the circular mitochondrial genome and capture the NuMTs comprising the mitochondrial genome’s control region. Potential NuMTs identified by Nuclear BLAST are then filtered and prioritised based on the E-Value (<1e-3) and query coverage (≥70%). The above Sniffles2 and Nuclear BLAST parameters were optimised using simulated data to minimise false positive rates. However, users can change these parameters by editing the config file provided by the tool. The final output of ANOMALY contains information about the nuclear breakpoint of the NuMT, including the size and location of the mitochondrial genome part integrated into the nuclear genome and a circos plot showing a visual representation of all integrated NuMTs (Fig. 1B).

### Alignment with the reference genome

Suppose the user chooses to input unaligned FASTQ files to the tool. In that case, the pipeline first aligns the input data to the human reference genome (including reference mitochondrial genome) using Minimap2 version 2.28 with parameters optimised for long reads (allowing soft clipping for supplementary alignments), followed by sorting and indexing of aligned data using SAMtools version 1.17 [19].

### Identification of potential NuMTs from structural variants

The sorted and indexed BAM file is then used to call Structural Variants (SVs) using Sniffles version 2.5 with parameters “sniffles –minsupport 4 –mapq 1 –minsvlen 24 –genotype-ploidy 2.” The insertion calls identified by Sniffles2 are then extracted and realigned with the concatenated reference mitochondrial FASTA using BLASTn version 2.15.0. The concatenated FASTA is generated using the concat command of SeqKit version 2.9.0. The main purpose of using the concatenated FASTA for the realignment is to capture NuMTs covering the control region of the mitochondrial genome. All the potential NuMTs captured by the realignment are then filtered based on E-value (<1e-3). The filtered NuMTs are concatenated if the distance between the calls is less than 10 bp using BEDtools version 2.30.0 [20]. The concatenated NuMTs are filtered based on query coverage (≥70%), resulting in the final NuMT calls from Structural Variants.

### Identification of potential NuMTs from supplementary alignments

In the previous study, it was reported that the size of the NuMTs can range from 39 bp to the complete length of the mitochondrial genome [10]. Larger NuMTs are difficult to capture as Structural Variants, as this often requires ultra-long reads as an input, which results in additional cost [21]. However, by leveraging the information stored as Supplementary alignments in the BAM file, this tool can capture full-length NuMTs. The pipeline first extracts the aligned reads from the nuclear genome having supplementary alignments with the mitochondrial reference genome. The neighbouring regions in the nuclear genome are concatenated using BEDtools if the distance between them is less than 100 bp. The potential nuclear breakpoints are then determined using the number of supporting reads (≥5). The final NuMT calls from supplementary alignments are then reported based on the breakpoints supported by at least five reads.

### Final NuMT call and visualisation

The NuMT calls from structural variants and supplementary alignments are concatenated, and unique calls are retained to report the final NuMTs. The final NuMT calls are then visualised as a circos plot using the R package circlise [22] version 0.4.16. The resulting circos plot is saved locally in PNG and SVG format. At the end of the run, the pipeline will produce one single circos plot annotating all the NuMTs called in all the input samples to give a more comprehensive overview of the NuMTs.

### Data simulation

To evaluate the pipeline’s performance, we simulated 546 unique NuMTs across 50 samples encompassing all autosomal and sex chromosomes, with NuMT size ranging from 30 bp to the full human mitochondrial genome length (16 569 bp). A bar plot showing the distribution of simulated NuMT length is provided in the Supplementary Fig. 1. The CHM13 human reference genome [22] was modified using the ‘mutate’ command in SeqKit version 2.9.0 to introduce the defined insertion sequences. Subsequently, PBSIM3 version 3.0.4 [23] was employed to generate sequencing data from the modified CHM13 genome using the ERRHMM-RSII error model. The resulting dataset had a 30X coverage depth and an average read length of 10 Kbp.

To benchmark ANOMALY against other NuMT calling methods, we also simulated the short-read sequencing datasets using the modified human reference genome. InSilicoSeq version 2.0.1 [24] was employed to generate sequencing data from the modified reference genome using the NovaSeq model with 30X nuclear sequencing depth.

ANOMALY and PALMER were evaluated using long-read simulated datasets, while the Wei *et al.* method and DINUMT were evaluated using short-read simulated datasets. For the final comparison, we applied the following quality filters: for DINUMT, we retained only calls tagged as “PASS” in the VCF file. We applied the authors’ recommended criteria for the Wei *et al.* method, requiring NuMTs to be supported by at least five discordant reads with defined nuclear and mitochondrial breakpoints. For PALMER, we included calls supported by at least five potential supporting reads.

### Pipeline optimisation

We optimised the classification performance of our pipeline by systematically evaluating four key parameters to maximise the F1 score. The parameters were: a) the minimum number of reads supporting an insertion, b) the Nucleotide Blast E-value threshold, c) the percentage of insert sequence coverage aligned to the reference mitochondrial genome, and d) the minimum number of reads supporting a NuMT call from supplementary alignments.

We tested 256 combinations of these parameters across 15 simulated datasets containing NuMTs of varying lengths (30 bp to the full mitochondrial genome). From the nine parameter sets that yielded the highest F1 score, we selected the one with the lowest thresholds for both insertion-supporting reads and supplementary reads supporting the NuMTs for the optimised pipeline {4, 0.001, 70, 5}. Detailed information regarding the parameter optimisation is provided in the Supplementary Table 2.

We next evaluated structural variant (SV) calling tools to optimise the pipeline. Based on a prior benchmarking study [25], which showed superior F1 scores for Sniffles2 and CuteSV version 2.1.2 [26], these two callers were selected for testing within the ANOMALY framework. On simulated datasets, ANOMALY with Sniffles2 outperformed the CuteSV-based version in both classification accuracy (F1 score: 0.994 vs 0.983) and computation efficiency (median execution time: 471.05 seconds vs 536.59 seconds and median peak memory usage: 0.55 GB vs 2.15 GB). Consequently, Sniffles2 was selected as the primary SV caller. Box plots showing the execution time and peak memory usage for Sniffles2 and CuteSV are shown in the Supplementary Fig. 2. Also, the detailed information regarding the NuMTs captured by the Sniffles2-based pipeline and the CuteSV-based pipeline is provided in the Supplementary Table 3.

Finally, to validate the design of our pipeline, we compared the performance of its three configurations: using only the Sniffles2 branch, only the supplementary alignment branch, and the full pipeline combining both. The combined approach achieved a superior F1 score (0.9967) compared to Sniffles2-only (0.9587) and supplementary-alignment-only (0.23) configurations (Supplementary Table 4). Complementary strengths of each method explain this performance gain: the Sniffles2 branch was more effective at detecting NuMTs shorter than 2500 bp, while the supplementary alignment branch excelled at capturing longer NuMTs (≥2500 bp). These results confirmed the advantage of integrating both approaches for comprehensive NuMT detection across all size ranges.

The final optimised pipeline was then used to detect NuMTs in the remaining 35 simulated samples, and the identified NuMTs were compared to the known true calls to establish true positives (TP), false positives (FP) and false negatives (FN). Finally, precision, recall and F1-score were calculated from these metrics to assess the overall performance of the pipeline.

## Results and discussion

### Performance evaluation

We benchmarked ANOMALY against three existing NuMT detection methods: the pipeline by W. Wei *et al.*, DINUMT, and PALMER. Performance was evaluated on 15 datasets comprising 170 unique NuMTs, using simulated short-read data from Wei *et al.* and DINUMT, and long-read data for ANOMALY and PALMER. ANOMALY achieved the highest classification performance, with an F1 score of 0.994, followed by PALMER (0.991), DINUMT (0.947), and Wei *et al.* (0.831). DINUMT was the fastest regarding computation efficiency, using only a single computation thread, followed by ANOMALY with 16 computational threads.

A detailed analysis revealed specific limitations in each comparator. The Wei *et al.* method failed to detect NuMTs shorter than 45 bp. DINUMT, while more sensitive, consistently mischaracterised NuMTs containing the control region (D-loop), accurately identifying the nuclear integration site but failing to determine the insert length and mitochondrial coordinates. ANOMALY overcame all these limitations, comprehensively characterising NuMTs without prior knowledge. Furthermore, ANOMALY detected all NuMTs identified by other tools and reported no additional false positives, confirming its status as the most suitable tool for NuMT discovery from long-read sequencing data (Table 1, Supplementary Table 5).

On the final 50 simulated datasets containing 546 unique NuMTs, ANOMALY successfully detected 540 NuMTs (TP) across all samples, achieving an overall precision of 1, a recall of 0.989 and an F1-score of 0.994. The six NuMTs (FN) missed by the pipeline had sizes varied between 32 bp and 901 bp, with more than 66% NuMTs less than 100 bp (Supplementary Table 6, Supplementary Fig. 3).

To establish the minimum sequencing depth required for comprehensive NuMT detection, we selected five datasets where ANOMALY had successfully identified all known NuMTs. We down-sampled these datasets to 10X, 15X, 20X and 25X coverage and re-ran ANOMALY. The results indicate a minimum depth of 25X is required, as ANOMALY captured all NuMTs at this depth but not consistently at lower depths (Supplementary Table 7).

Finally, to assess the generalisability of ANOMALY, we evaluated its performance on simulated datasets representing four major human mitochondrial haplogroups (B, L0, L3 and M). We analysed 20 datasets (five per haplogroup) and found that ANOMALY successfully captured most NuMTs across all haplogroups. A single NuMT was missed in one dataset due to insufficient supporting reads from supplementary alignments (Supplementary Table 8). This result demonstrates that ANOMALY’s performance is robust across diverse human ancestries, making it suitable for analysing datasets from varied populations.

### Resolution of complex NuMT present in real-world data

To validate ANOMALY on real-world data, we analysed six publicly available datasets with matched long-read [27] and short-read sequencing data [Bioproject ID: PRJEB3381]. We compared ANOMALY’s results against PALMER from long-read data and the W. Wei *et al.* pipeline and DINUMT from short-read data.

ANOMALY demonstrated superior performance, capturing NuMTs supported by either supplementary alignments to the mitochondrial genome or high-confidence sniffles insertions, mapping to the mitochondrial genome (Table 2). However, it missed NuMT, located at chromosome 11:101583361, captured by the short-read callers. While PALMER captured more NuMTs as compared to all three methods, an inspection of these results revealed that the majority of the calls were likely to be false positives due to the lack of evidence of mitochondrial origin (Supplementary Table 9C).

**Table 2.** Performance comparison of NuMT detection tools on real-world datasets

Sample	Method	CalledNuMTs	ExecutionTime (Seconds)	PeakMemoryUsage (GB)	AverageAutosomalCoverage
NA12877	ANOMALY	19	2709.63	12.0	105.41
NA12878	ANOMALY	16	2320.37	9.44	102.29
NA12889	ANOMALY	20	1646.13	7.09	59.87
NA12890	ANOMALY	16	1498.66	6.58	53.58
NA12891	ANOMALY	22	1649.30	6.28	62.21
NA12892	ANOMALY	18	1412.55	6.18	57.09
NA12877	DINUMT	8	487.41	0.02	53.94
NA12878	DINUMT	11	1991.14	0.02	51.18
NA12889	DINUMT	6	1720.75	0.02	29.43
NA12890	DINUMT	7	1406.58	0.02	45.82
NA12891	DINUMT	8	2779.7	0.02	51.56
NA12892	DINUMT	5	2043.82	0.02	54.45
NA12877	W. Wei *et al.*	6	6743.73	20.85	53.94
NA12878	W. Wei *et al.*	9	9330.22	20.17	51.18
NA12889	W. Wei *et al.*	4	10986.52	14.90	29.43
NA12890	W. Wei *et al.*	8	13873.72	19.37	45.82
NA12891	W. Wei *et al.*	7	19250.5	27.35	51.56
NA12892	W. Wei *et al.*	7	17910.88	21.69	54.45
NA12877	PALMER	32	94757.44	51.11	105.41
NA12878	PALMER	25	77019.78	33.44	102.29
NA12889	PALMER	26	34704.67	23.26	59.87
NA12890	PALMER	21	46174.59	18.37	53.58
NA12891	PALMER	27	54802.64	33.58	62.21
NA12892	PALMER	19	46965.49	18.02	57.09

In contrast, the short-read-based methods exhibited significant limitations: the Wei *et al.* method missed 58 NuMT calls, while DINUMT produced one false positive and missed 58 calls. Detailed results are provided in Supplementary Tables 9 and 10, with IGV screenshots for the NA12878 cell line in Supplementary Fig. 4 and for the remaining samples in the ANOMALY GitHub repository [https://github.com/Nirmal2310/ANOMALY].

Furthermore, ANOMALY resolved a critical discrepancy in a complex NuMT event in all samples except the NA12877 cell line. This event was located at chromosome 5:32,452,190. Short-read callers interpreted this as a single 2,116 bp insertion, while PALMER did not capture this NuMT across all five samples. Still, ANOMALY’s analysis of long-read data revealed two separate mitochondrial segments totalling 290 bp, separated by a 1,965 bp gap in the mitochondrial genome (Supplementary Table 10, Supplementary Fig. 5). This short-read-based caller’s error was attributed to discordant read mapping; some discordant reads appeared to align with the first mitochondrial region. In contrast, others aligned to the second, resulting in an overestimation of the NuMT length. A schematic representation of this phenomenon is provided in Supplementary Fig. 6.

This study presents ANOMALY, a novel bioinformatics pipeline for comprehensive detection of nuclear mitochondrial DNA segments (NuMTs) from long-read sequencing data. While existing tools are incompatible with long reads or, like PALMER, not optimised to capture broad ranges of NuMTs, ANOMALY overcomes these limitations. It integrates two complementary approaches: a structural variant caller optimised for shorted NuMTs and a supplementary alignment method for longer inserts, ensuring high sensitivity across all insertion sizes. Benchmarks demonstrate that ANOMALY achieves superior classification accuracy, successfully resolves complex NuMT events mischaracterised by short-read callers, and performs robustly across diverse human mitochondrial haplogroups. The pipeline provides standardised, ready-to-use outputs, facilitating direct downstream analysis, establishing it as a versatile tool for the community.

## Conclusion

ANOMALY is a Snakemake pipeline for accurately identifying NuMTs from long-read sequencing data. It is an easy-to-install end-to-end pipeline that does not require extensive experience with bioinformatics. In this article, we have displayed the use case of this pipeline on human data; however, this pipeline can be adapted to any organism as long as the long-read data is available. This pipeline also provides a publication-ready plot visualising NuMTs in the samples, which can be directly adapted to the research article.

## Supplementary Material

lqag014_Supplemental_Files

## Data Availability

Project Name: ANOMALY. Project Home Page: https://github.com/Nirmal2310/ANOMALY Archived at: https://doi.org/10.5281/zenodo.18335914 Operating system(s): Linux. Programming Language: Snakemake (Python), Bash, R. Other requirements: Dependencies installed via conda and pip. Licence: GNU GPLv3.
