# Supplementary material for: ANOMALY: a Snakemake pipeline for identifying NuMTs from long-read sequencing data
Source: NAR Genom Bioinform. 2026 Feb 4;8(1):lqag014. doi: 10.1093/nargab/lqag014 (PMC12869244; doi:10.1093/nargab/lqag014)
Supplement: lqag014_Supplemental_Files [file lqag014_supplemental_files.zip › Supplementary Figures.docx]

**
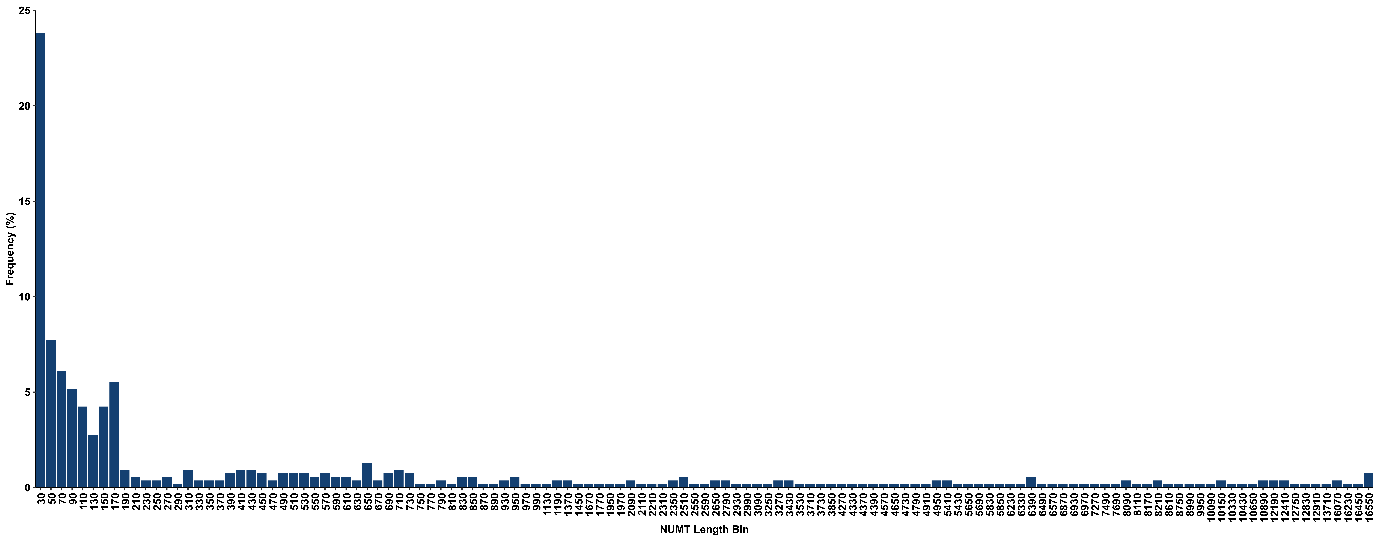
**

**Supplementary Figure 1:** Bar plot showing the distribution of NuMT length simulated across 50 datasets, each bin has a size of 20 bp. The NuMTs ranged from 30bp to full length mitochondrial genome size (16,569 bp), with majority of NuMTs ranging from 30-50 bp.

**
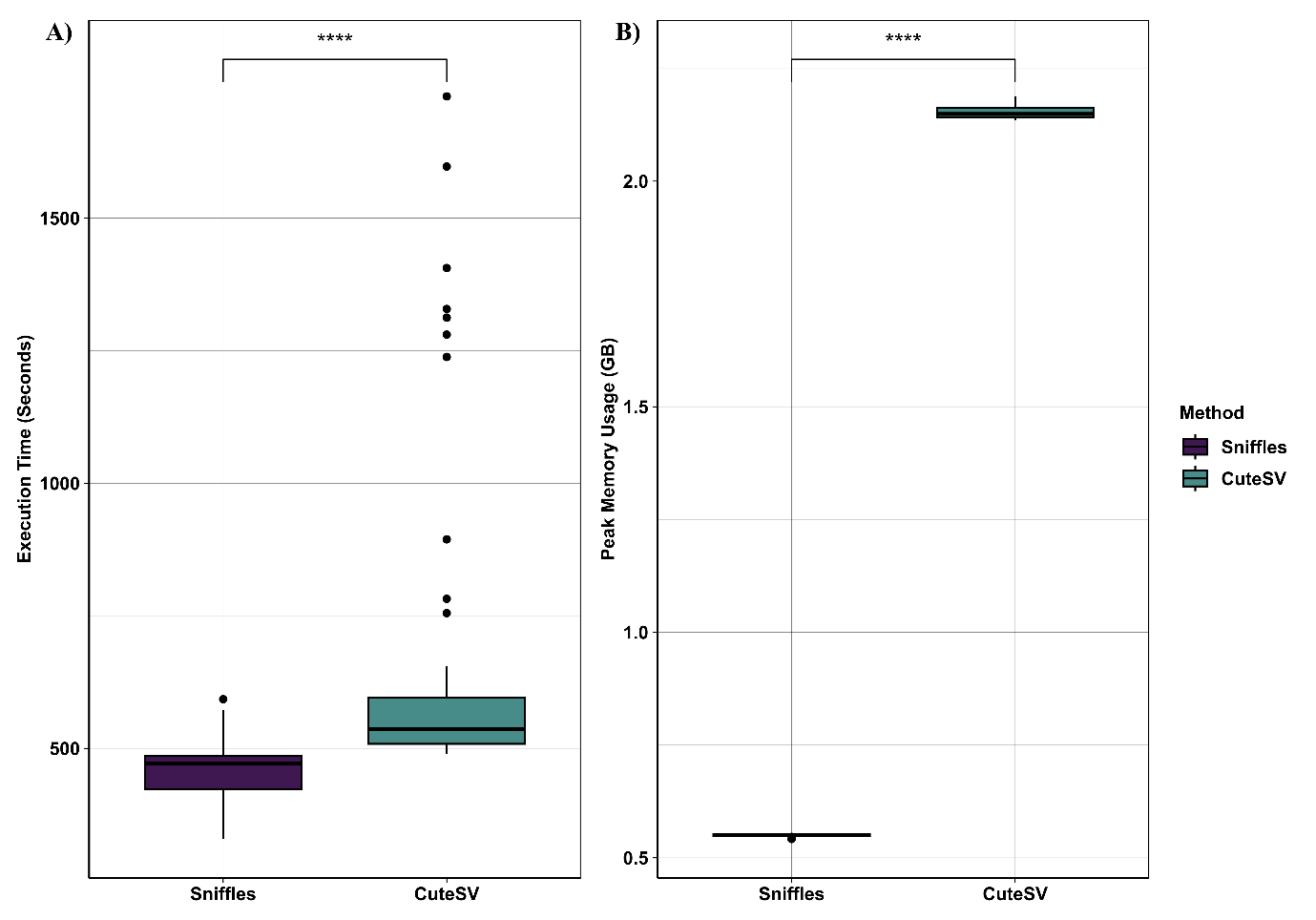
**

**Supplementary Figure 2:** Comparison of runtime performance metrics between the SV callers Sniffles2 and CuteSV. **A)** Boxplot showing the comparison of execution time (seconds) between Sniffles2 and CuteSV. **B)** Boxplot showing the comparison of peak memory usage (GB) between Sniffles2 and CuteSV. `****` indicates p-value ≤ 0.0005; p-value was calculated using the Wilcoxon ranked-sum test.

Sniffles2 outperformed CuteSV both in terms of execution time and peak memory usage aligning with our decision of using Sniffles2 as the main SV caller.

**
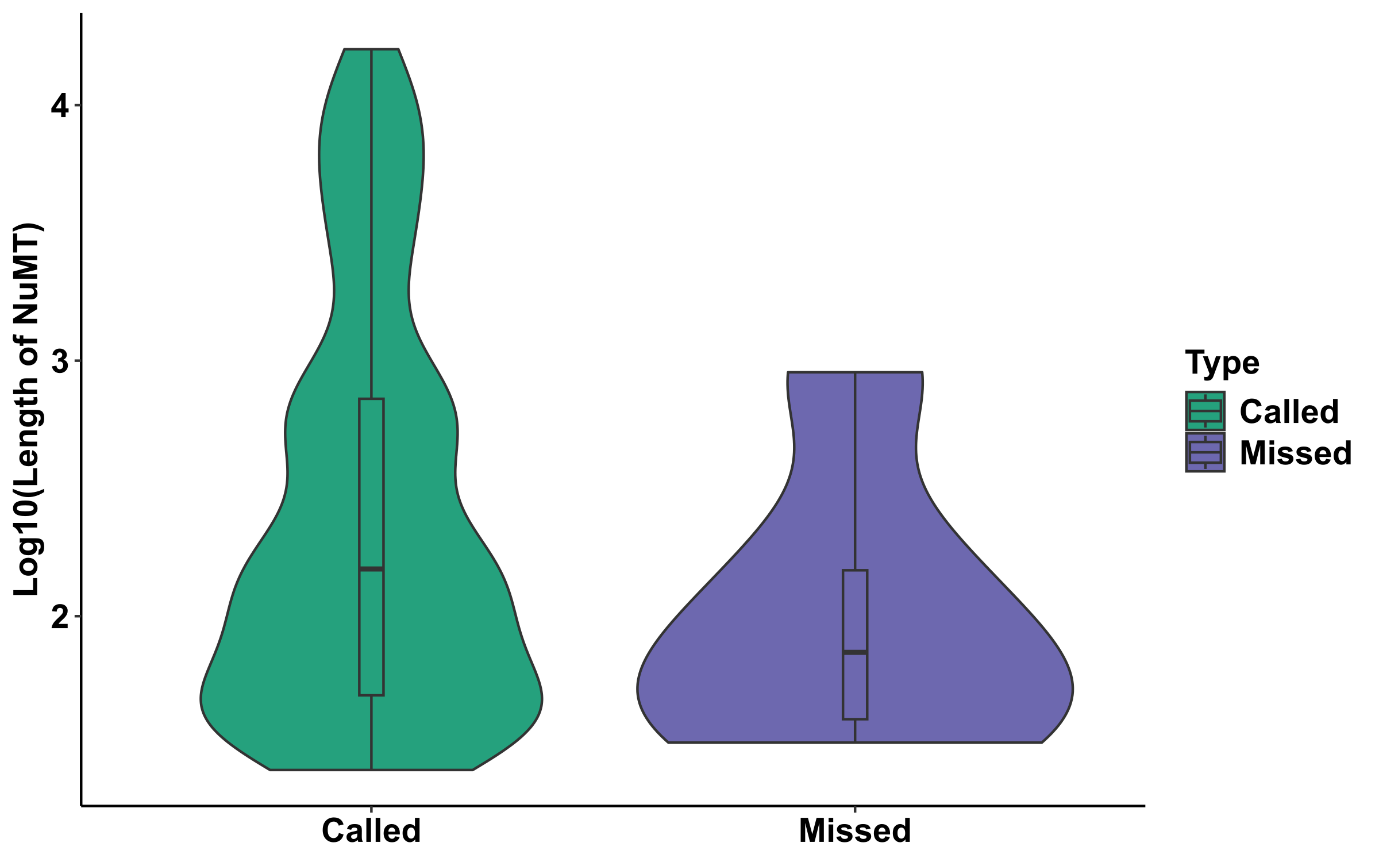
**

**Supplementary Figure 3:** Violin Plot showing the length distribution of NuMTs called and missed by the pipeline. 4 out of 6 missed NuMTs had a length of less than 100 bp.


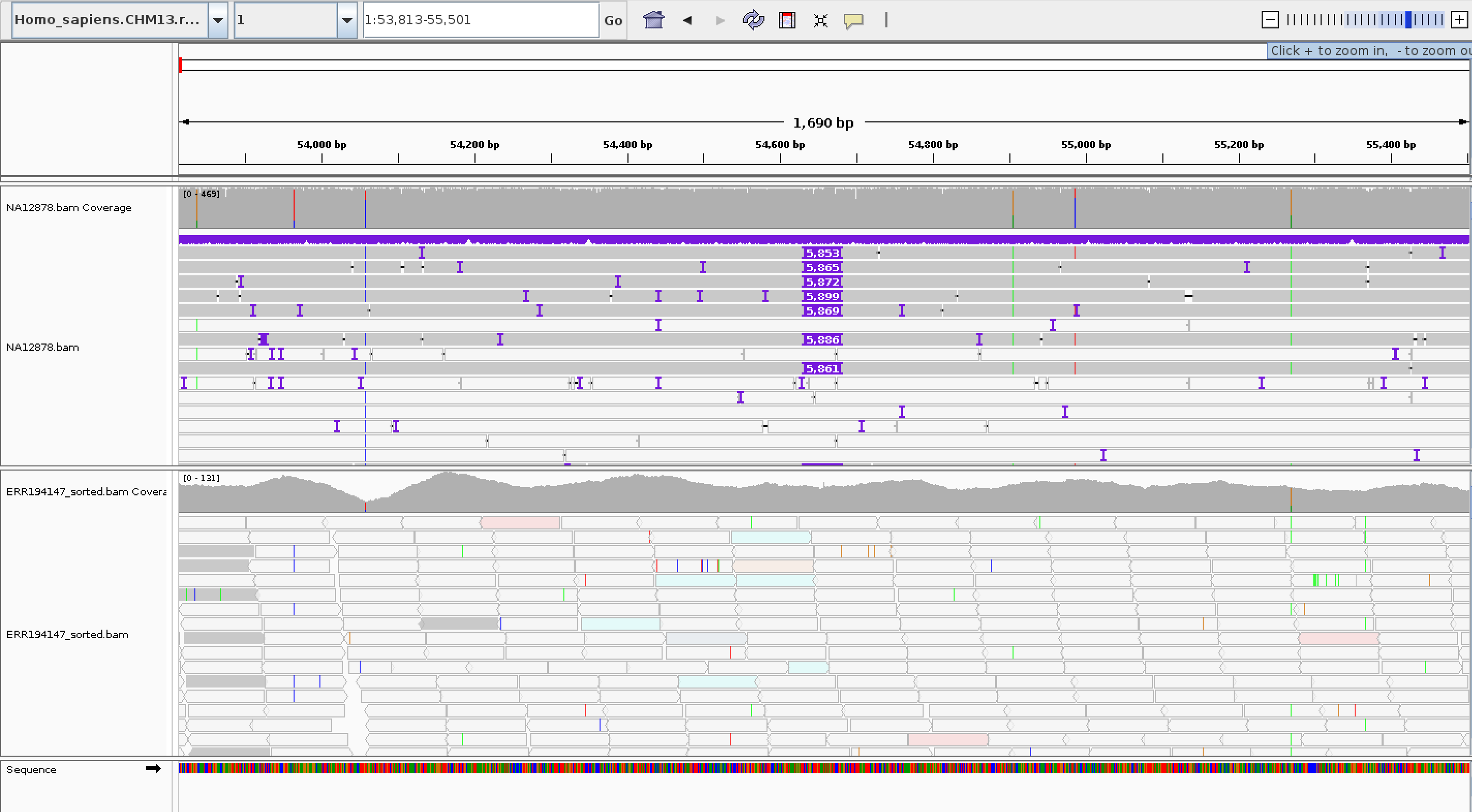


**Supplementary Figure 4(A):** IGV Screenshot of an NuMT called by ANOMALY only. The NuMT is shown as an insertion in Long-read sequencing data. This NuMT is completely missed by DINUMT and Wei et al. method.


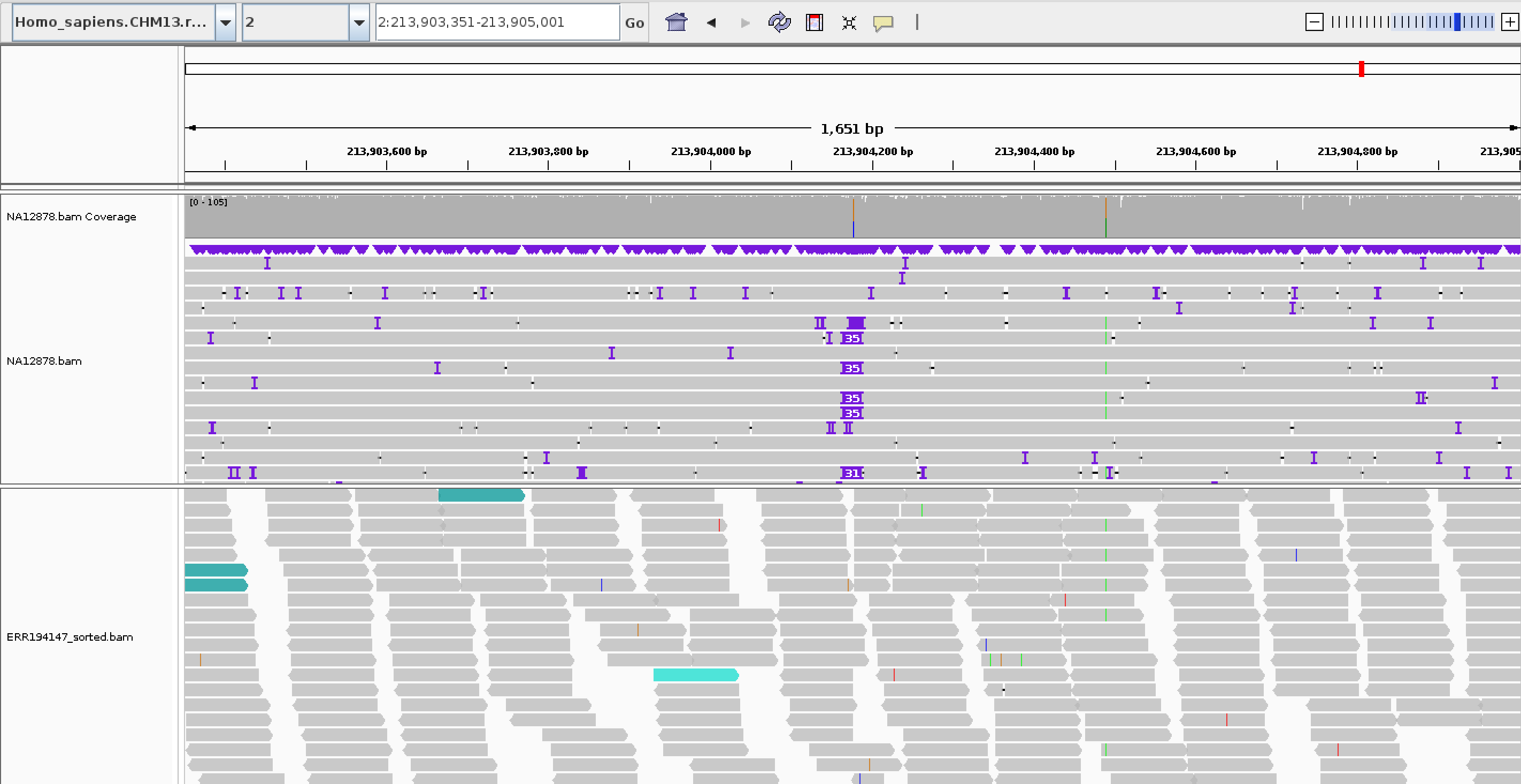


**Supplementary Figure 4(B):** IGV Screenshot of an NuMT called by ANOMALY and DINUMT but missed by Wei et al due to insufficient discordant reads supporting the call.


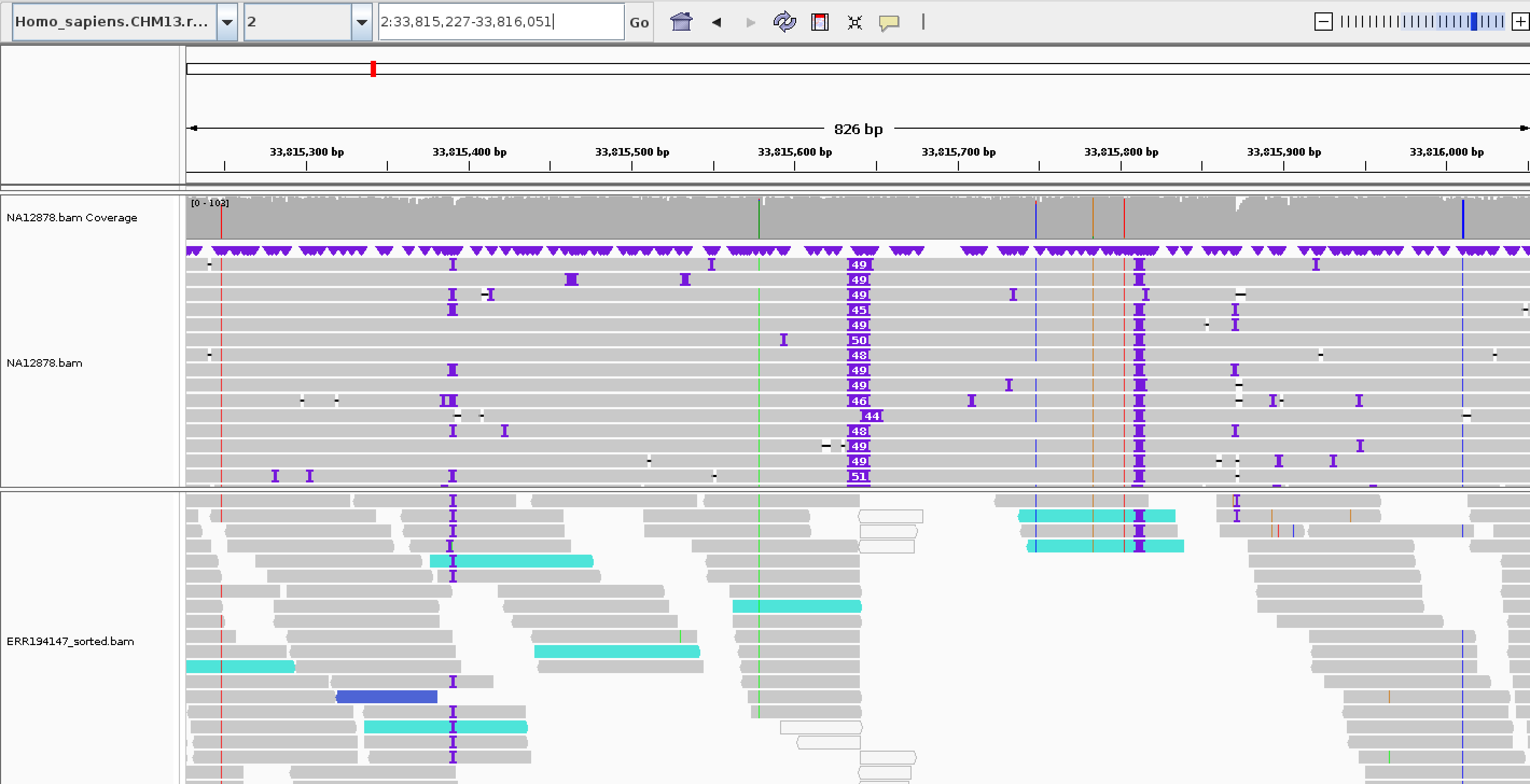


**Supplementary Figure 4(C):** IGV Screenshot of an NuMT called by all three methods. The NuMT is shown as an insertion in Long-read sequencing data and as discordant reads mapping to mitochondrial genome (turquoise colour) in Short-read sequencing data.

**
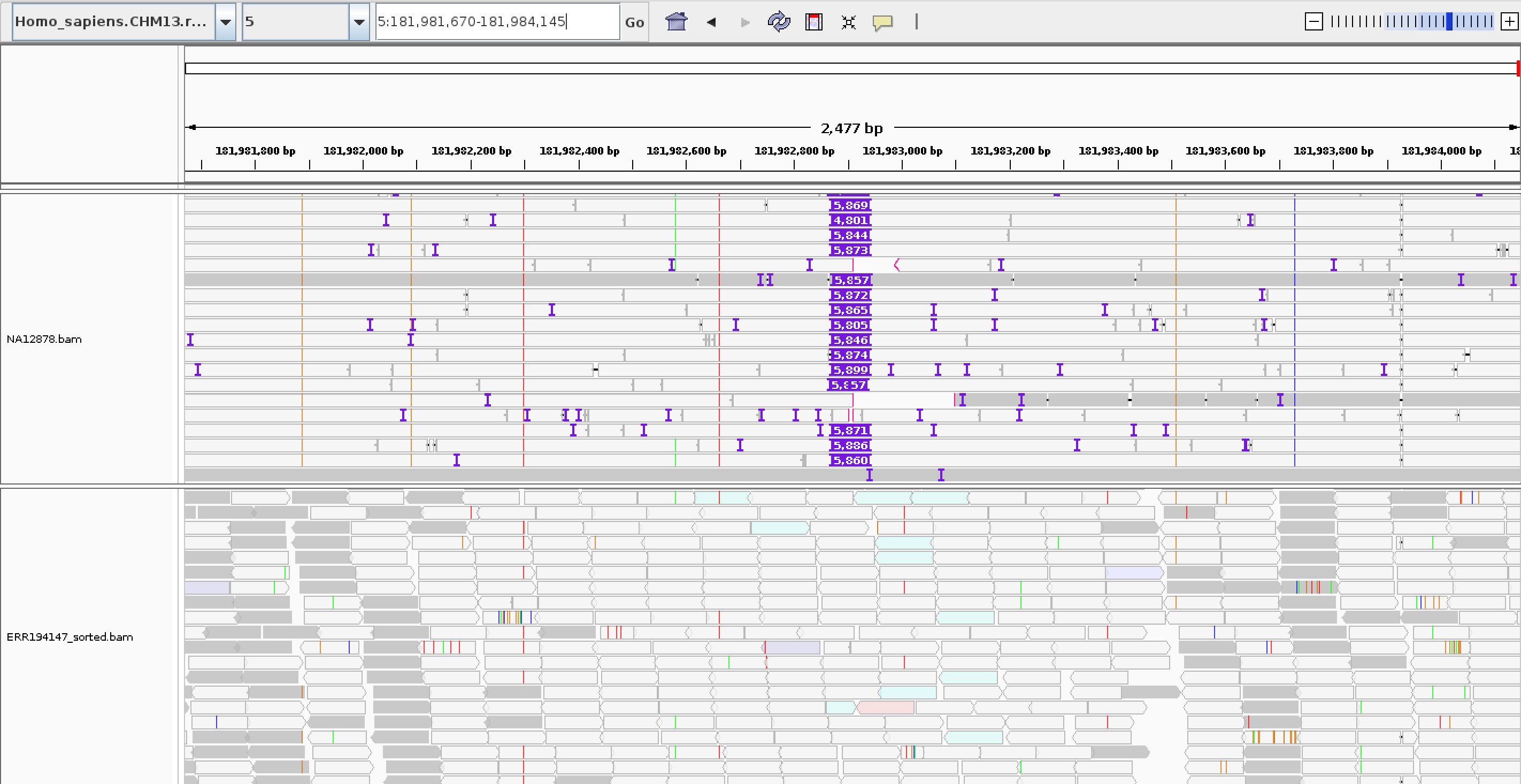
**

**Supplementary Figure 4(D):** IGV Screenshot of an NuMT called by ANOMALY only. The NuMT is shown as an insertion in Long-read sequencing data. This NuMT is completely missed by DINUMT and Wei et al. method.

**
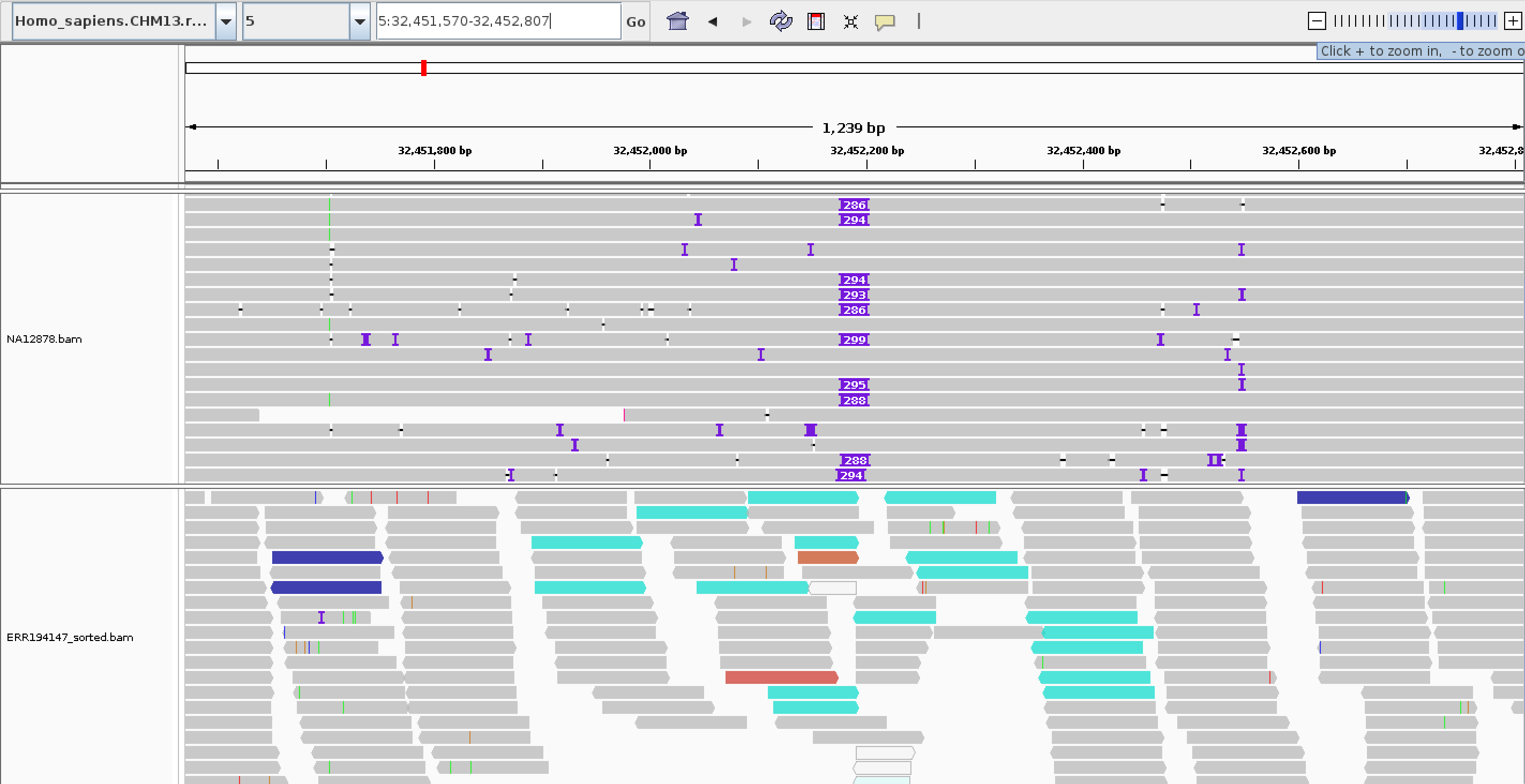
**

**Supplementary Figure 4(E):** IGV Screenshot of an NuMT called by all three methods. The NuMT is shown as an insertion in Long-read sequencing data and as discordant reads mapping to mitochondrial genome (turquoise colour) in Short-read sequencing data.

**
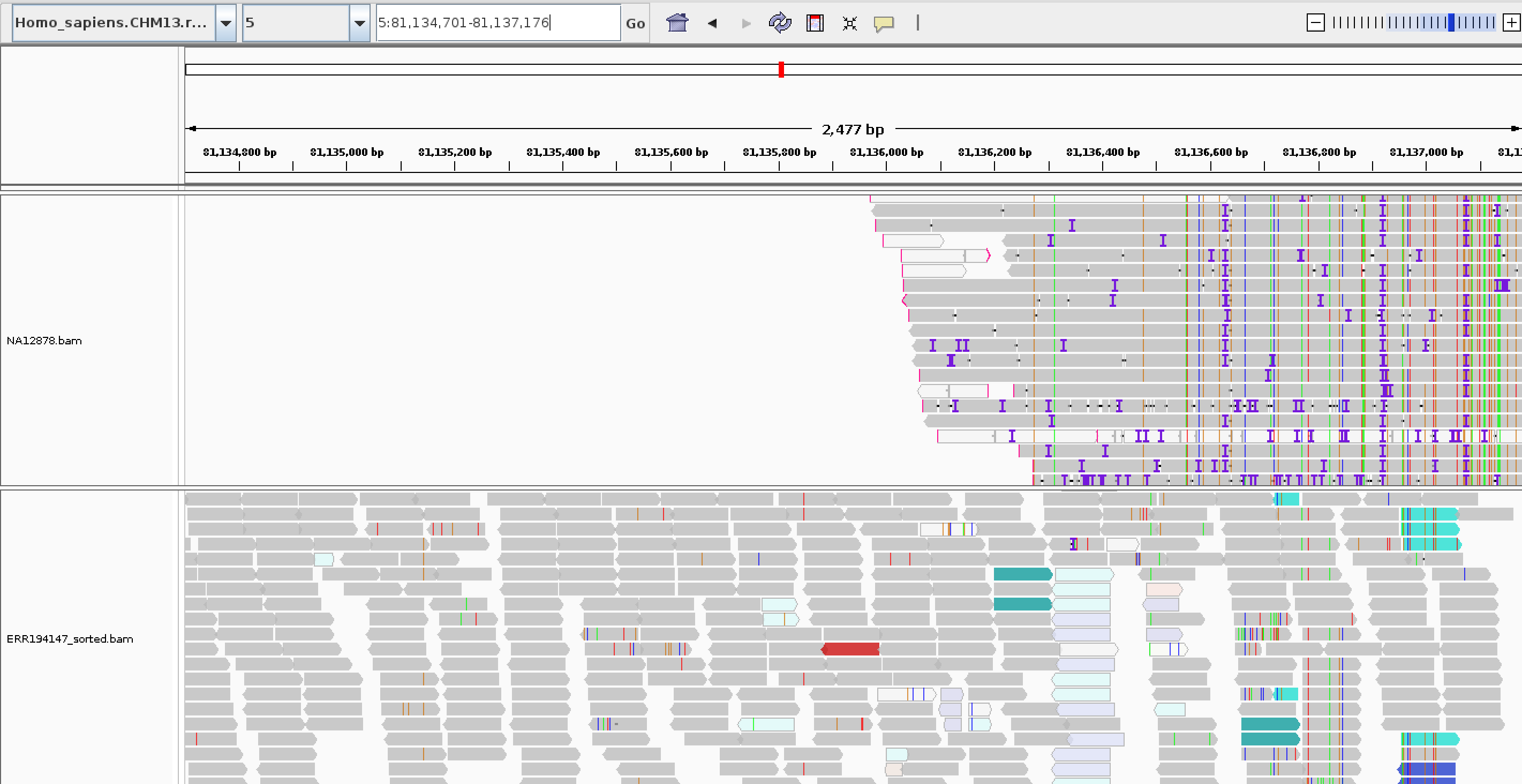
**

**Supplementary Figure 4(F):** IGV Screenshot of an NuMT called by ANOMALY and Wei et al and missed by DINUMT.

**
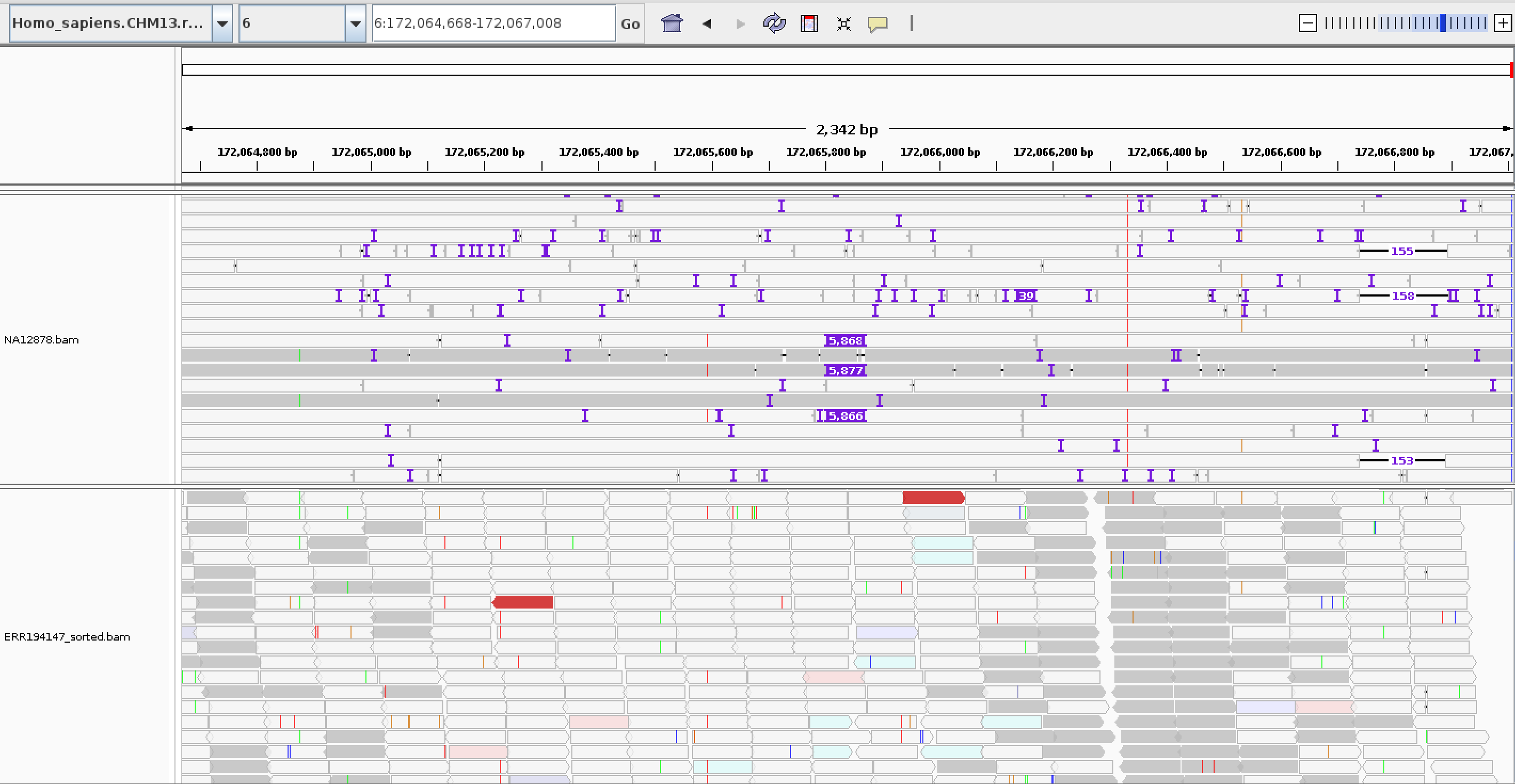
**

**Supplementary Figure 4(G):** IGV Screenshot of an NuMT called by ANOMALY only. The NuMT is shown as an insertion in Long-read sequencing data. This NuMT is completely missed by DINUMT and Wei et al. method.

**
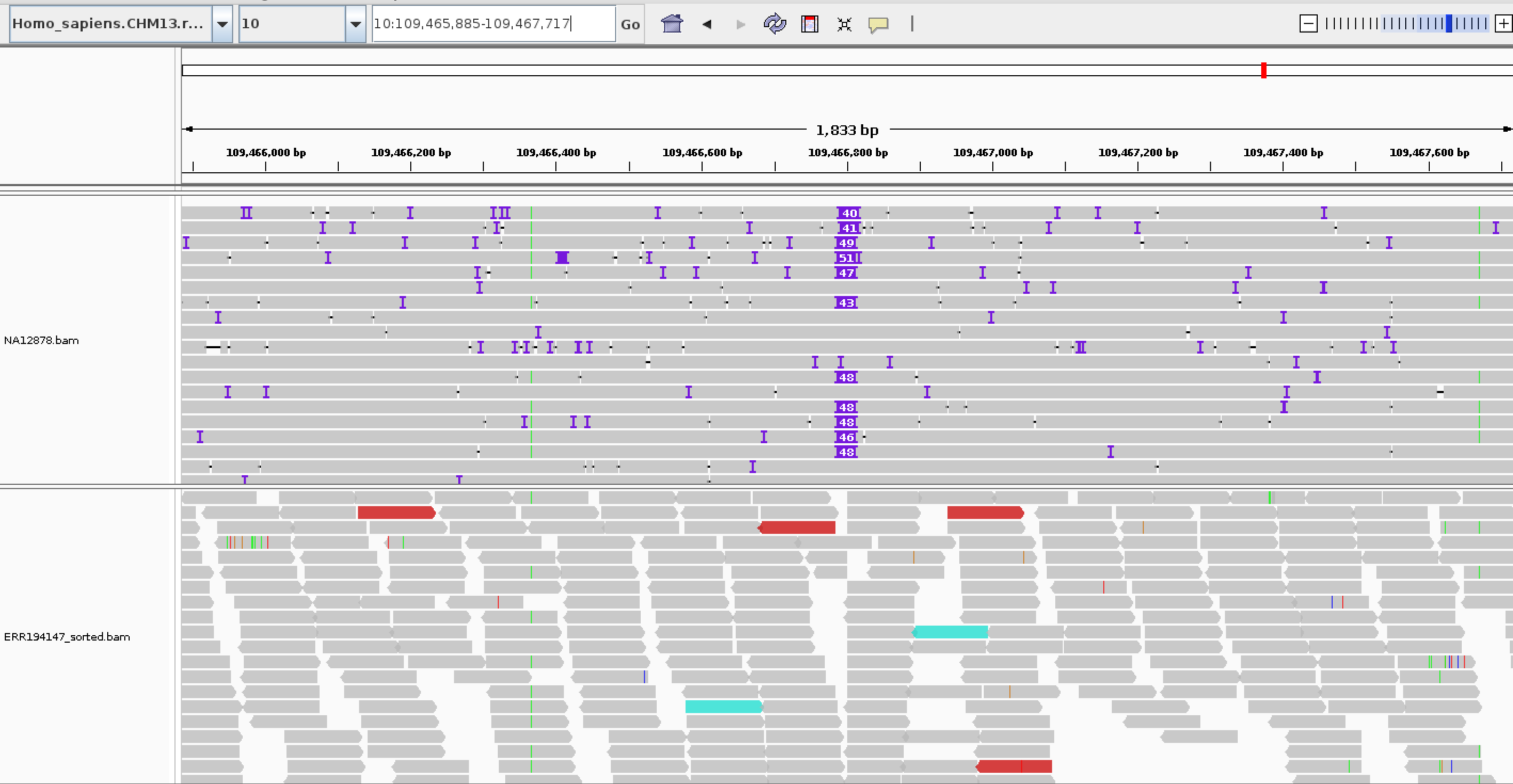
**

**Supplementary Figure 4(H):** IGV Screenshot of an NuMT called by all three methods. The NuMT is shown as an insertion in Long-read sequencing data and as discordant reads mapping to mitochondrial genome (turquoise colour) in Short-read sequencing data.

**
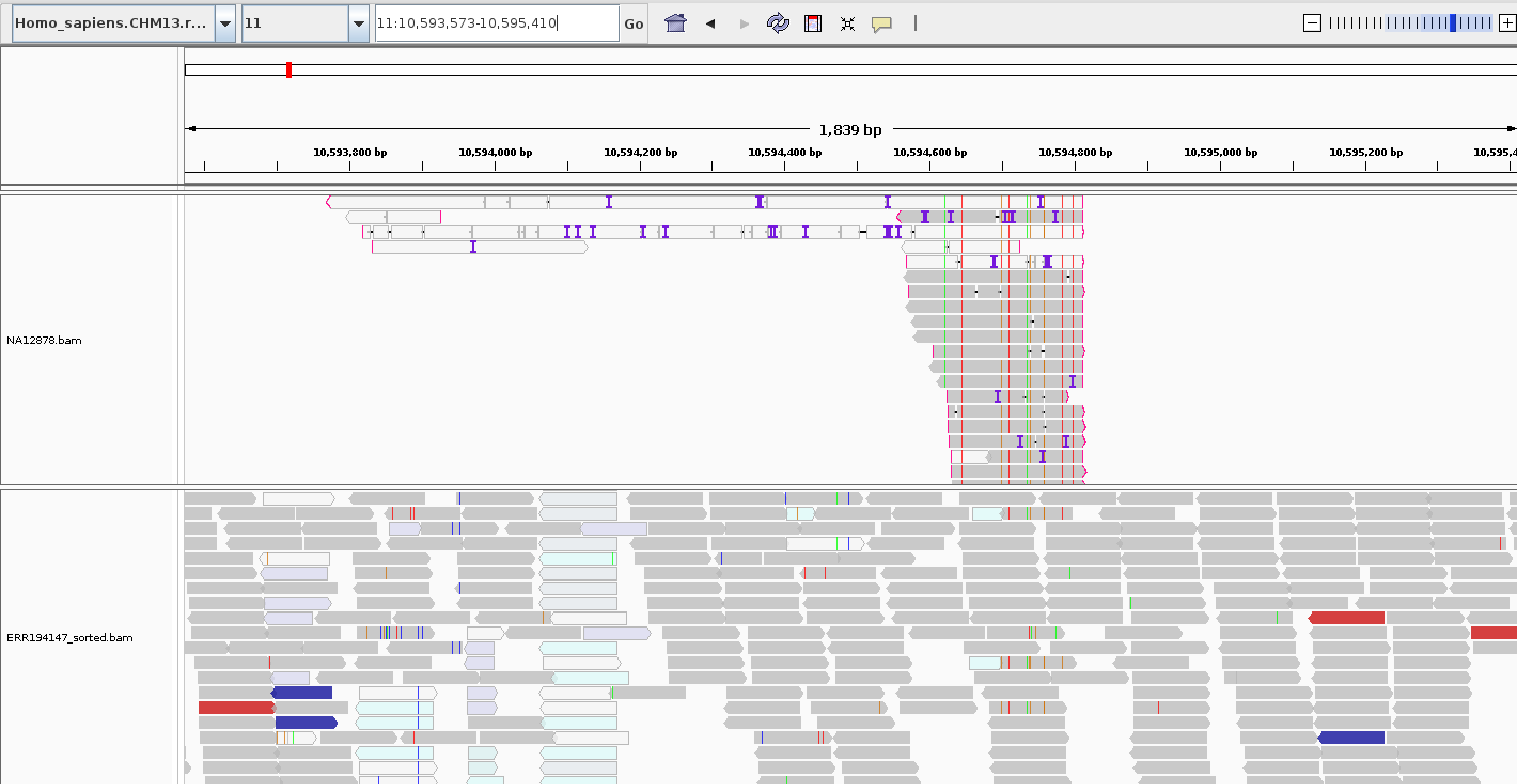
**

**Supplementary Figure 4(I):** IGV Screenshot of an NuMT called by ANOMALY and Wei et al and missed by DINUMT.

**
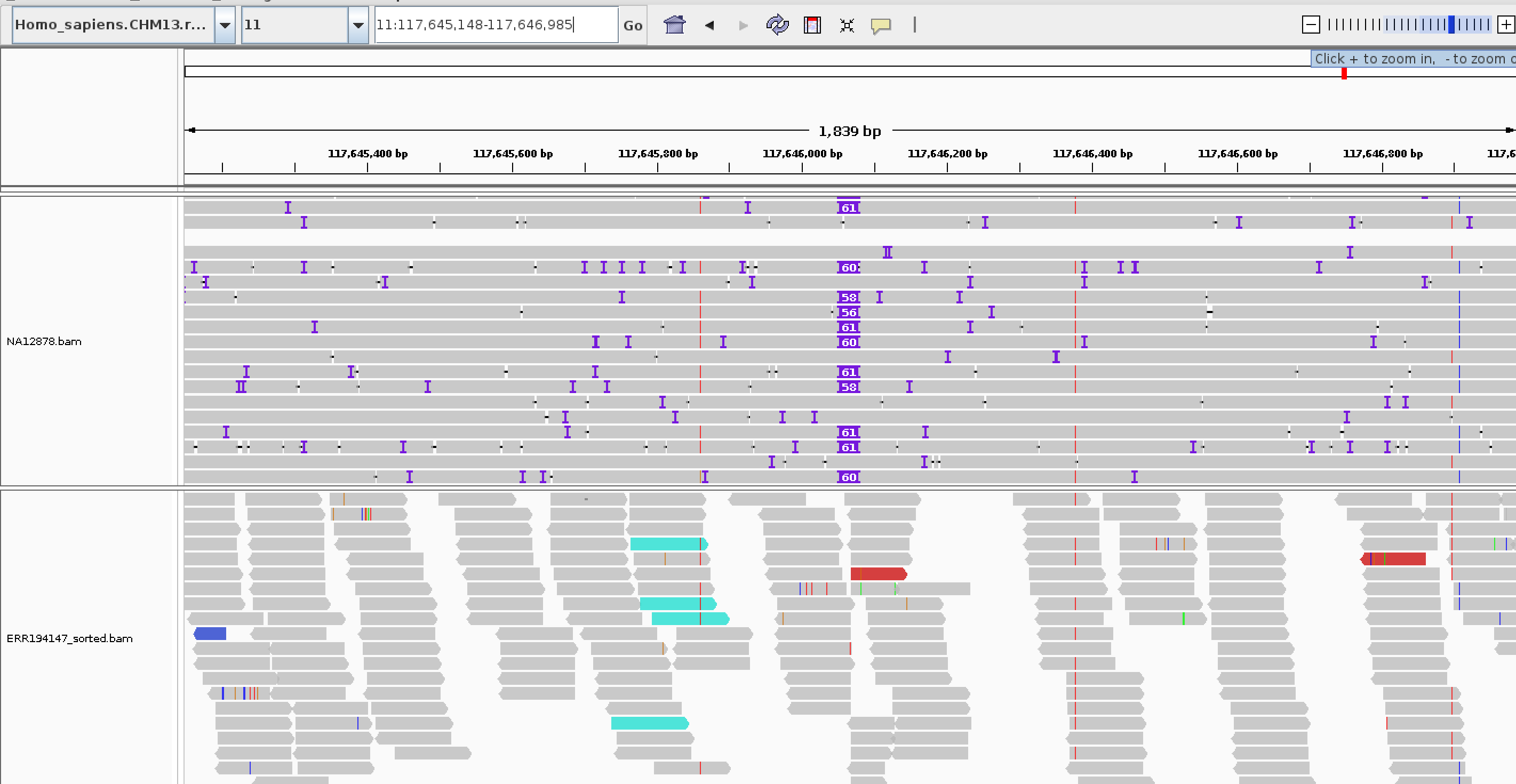
**

**Supplementary Figure 4(J):** IGV Screenshot of an NuMT called by all three methods. The NuMT is shown as an insertion in Long-read sequencing data and as discordant reads mapping to mitochondrial genome (turquoise colour) in Short-read sequencing data.

**
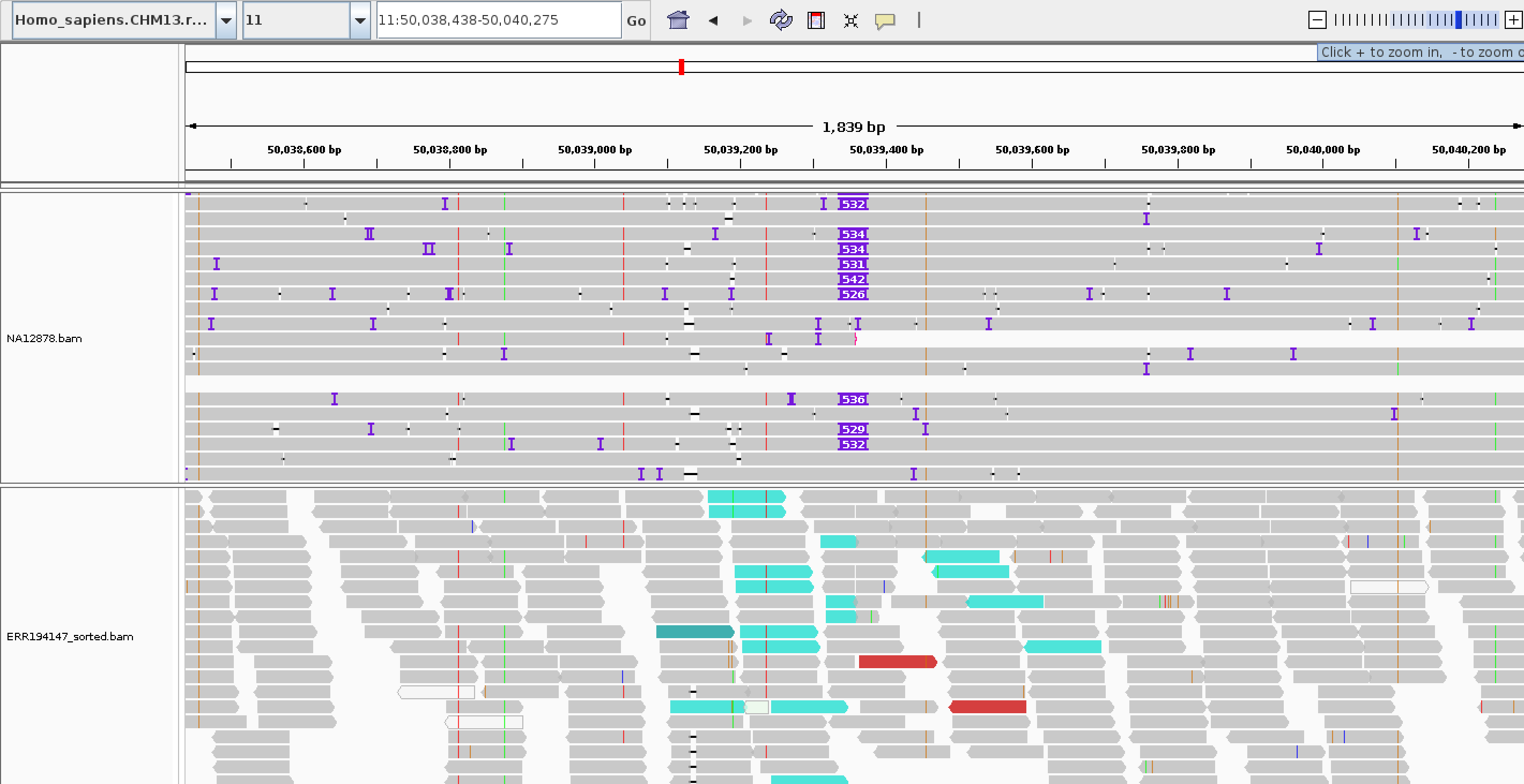
**

**Supplementary Figure 4(K):** IGV Screenshot of an NuMT called by all three methods. The NuMT is shown as an insertion in Long-read sequencing data and as discordant reads mapping to mitochondrial genome (turquoise colour) in Short-read sequencing data.


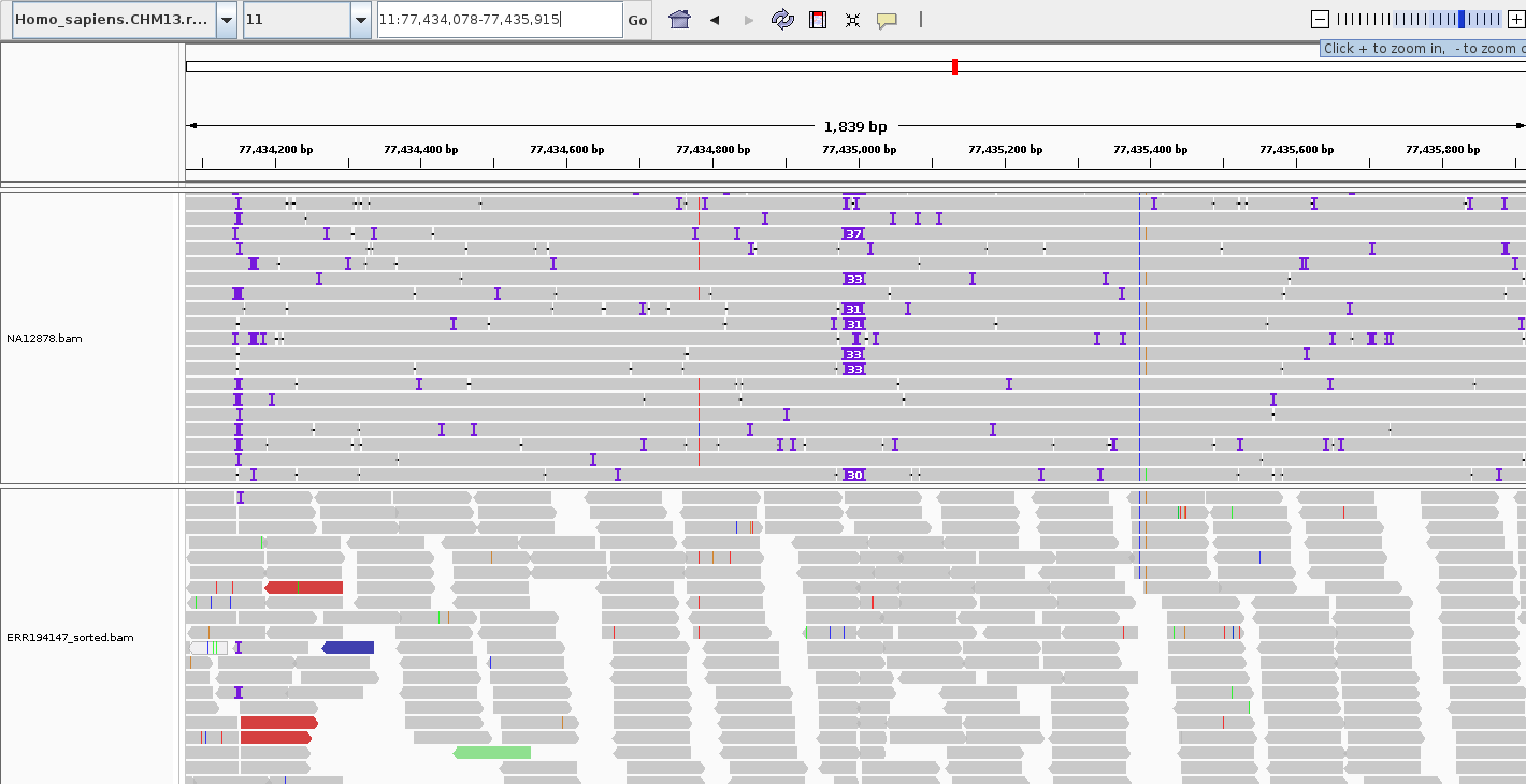


**Supplementary Figure 4(L):** IGV Screenshot of an NuMT called by ANOMALY and DINUMT but missed by Wei et al due to insufficient discordant reads supporting the call.

**
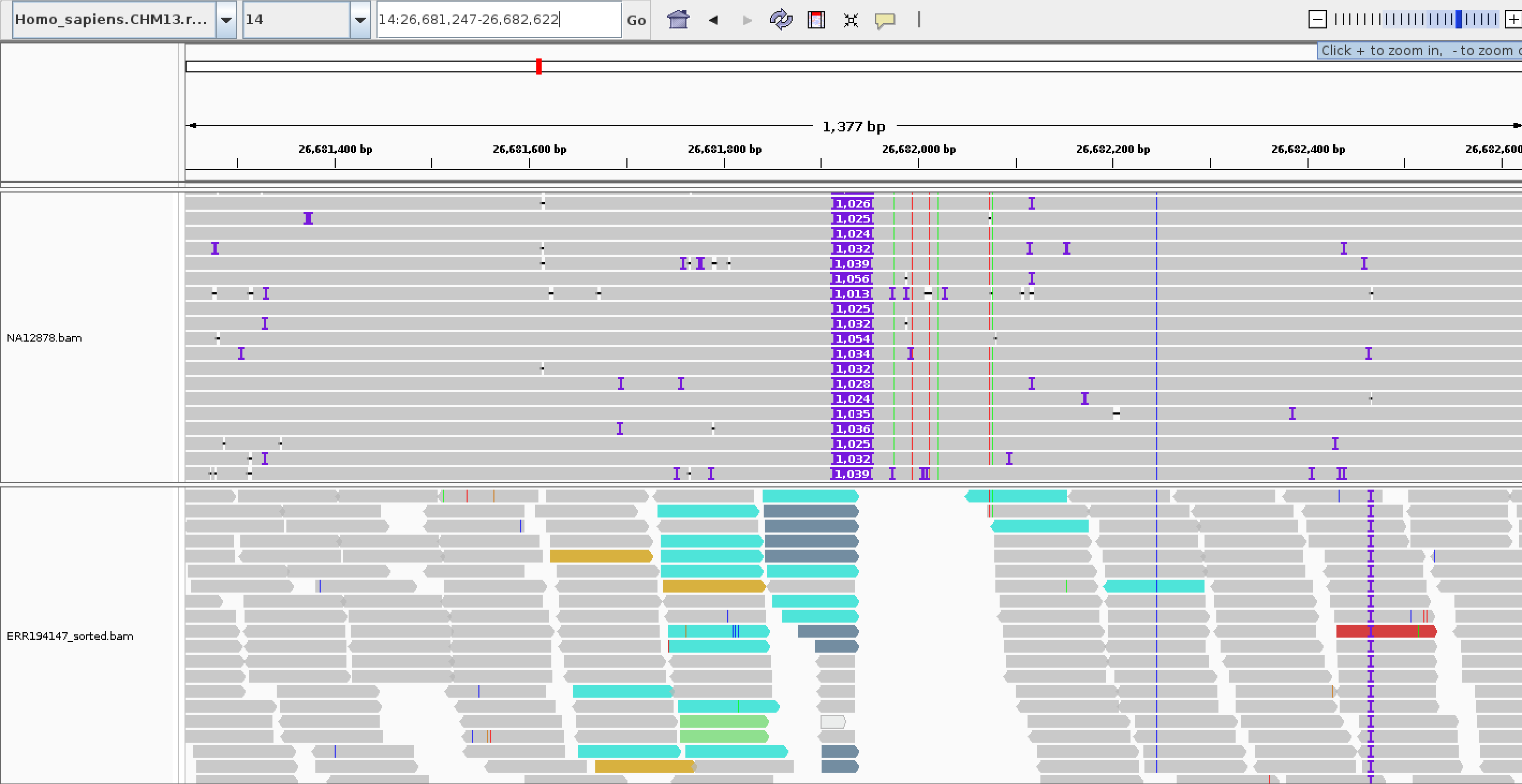
**

**Supplementary Figure 4(M):** IGV Screenshot of an NuMT called by ANOMALY and DINUMT but missed by Wei et al method due to not enough reads supporting the call.

**
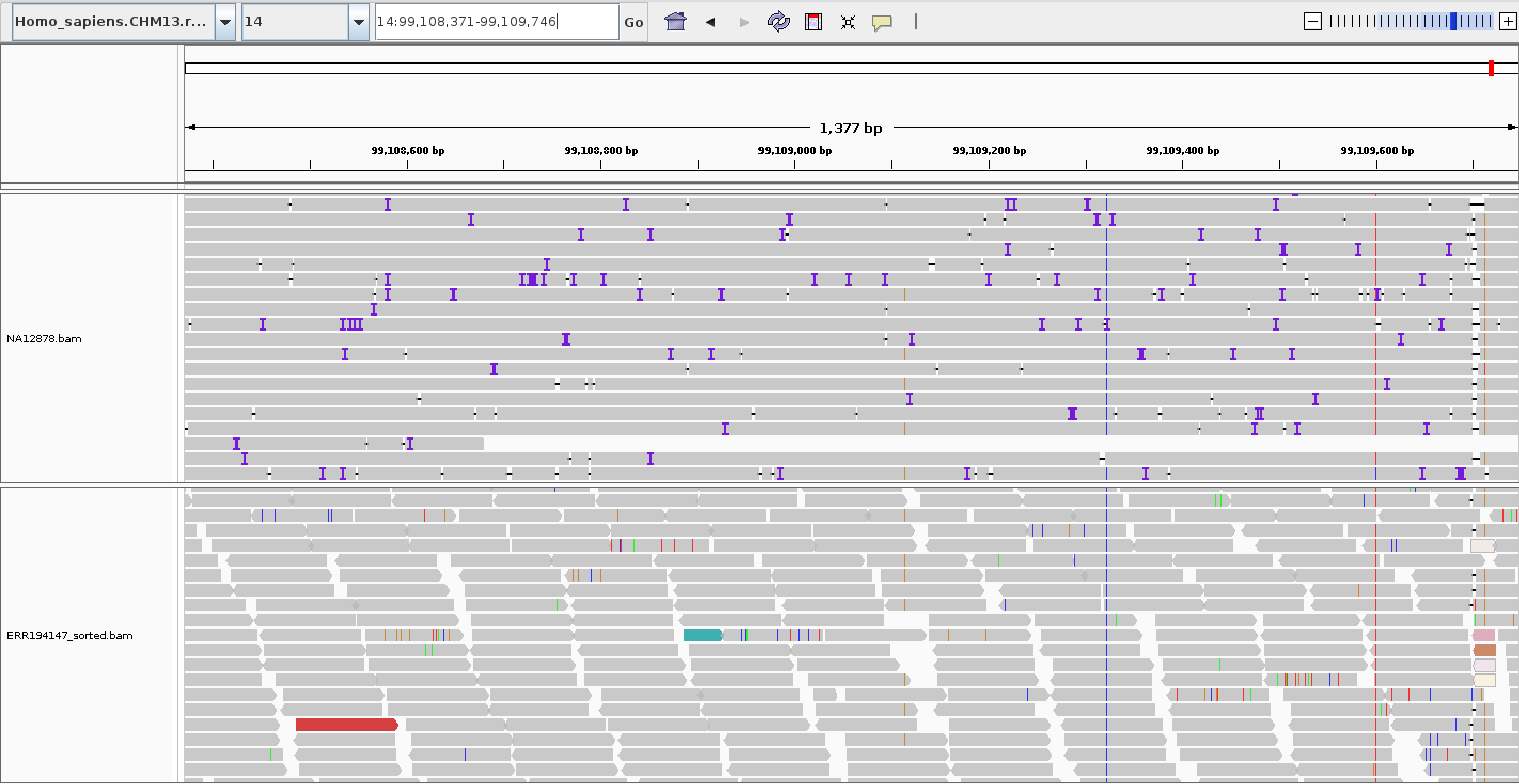
**

**Supplementary Figure 4(N):** IGV Screenshot of an NuMT called by DINUMT only. This call is a False-Positive as there are no signatures of insertions in both long-read as well as short-read sequencing data.

**
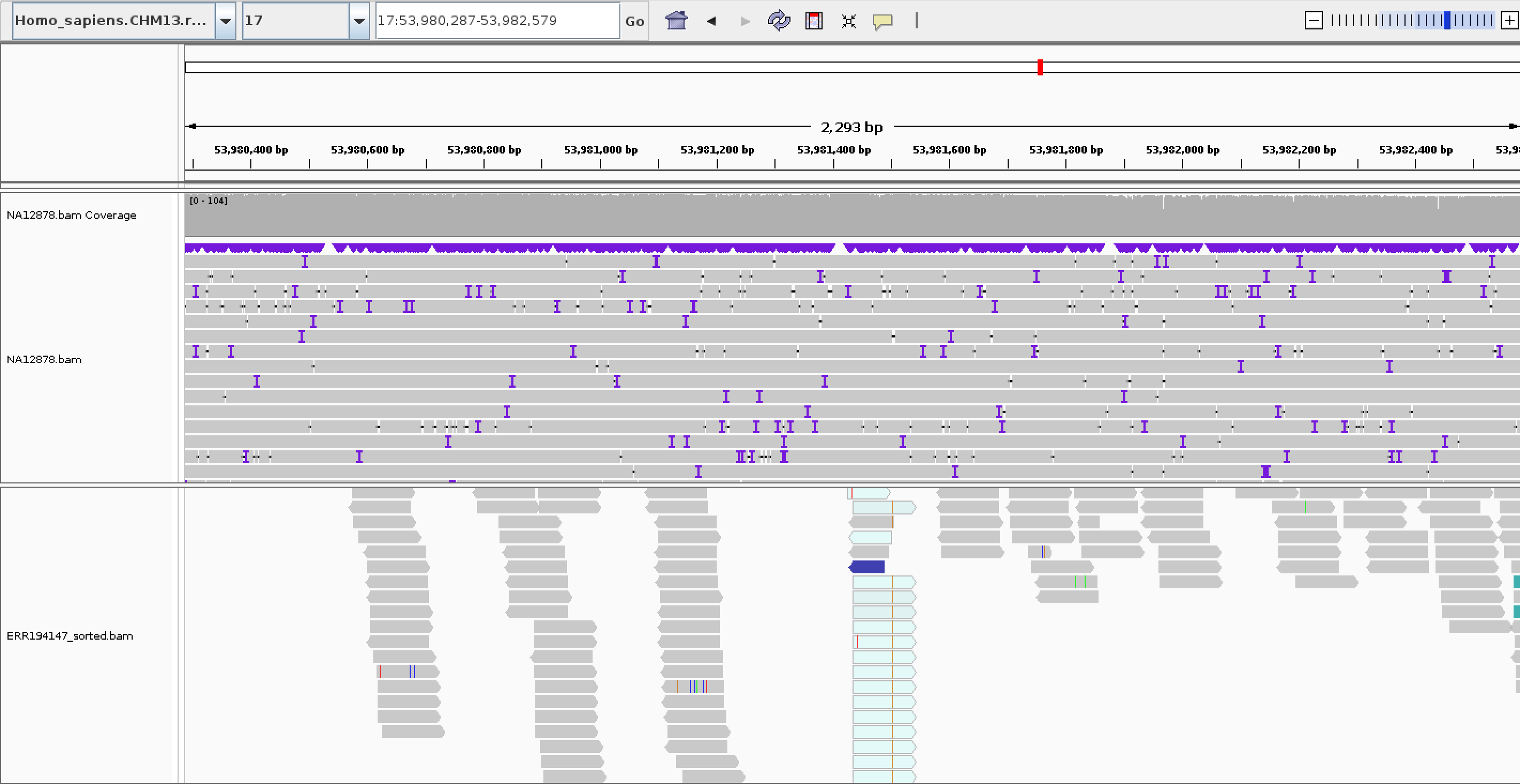
**

**Supplementary Figure 4(O):** IGV screenshot of an NuMT called by only Wei et al. method. The short-read sequencing data showed signs of discordant reads mapping to mitochondrial genome (turquoise colour). However, no insertion calls were present in the long-read sequencing data.

**
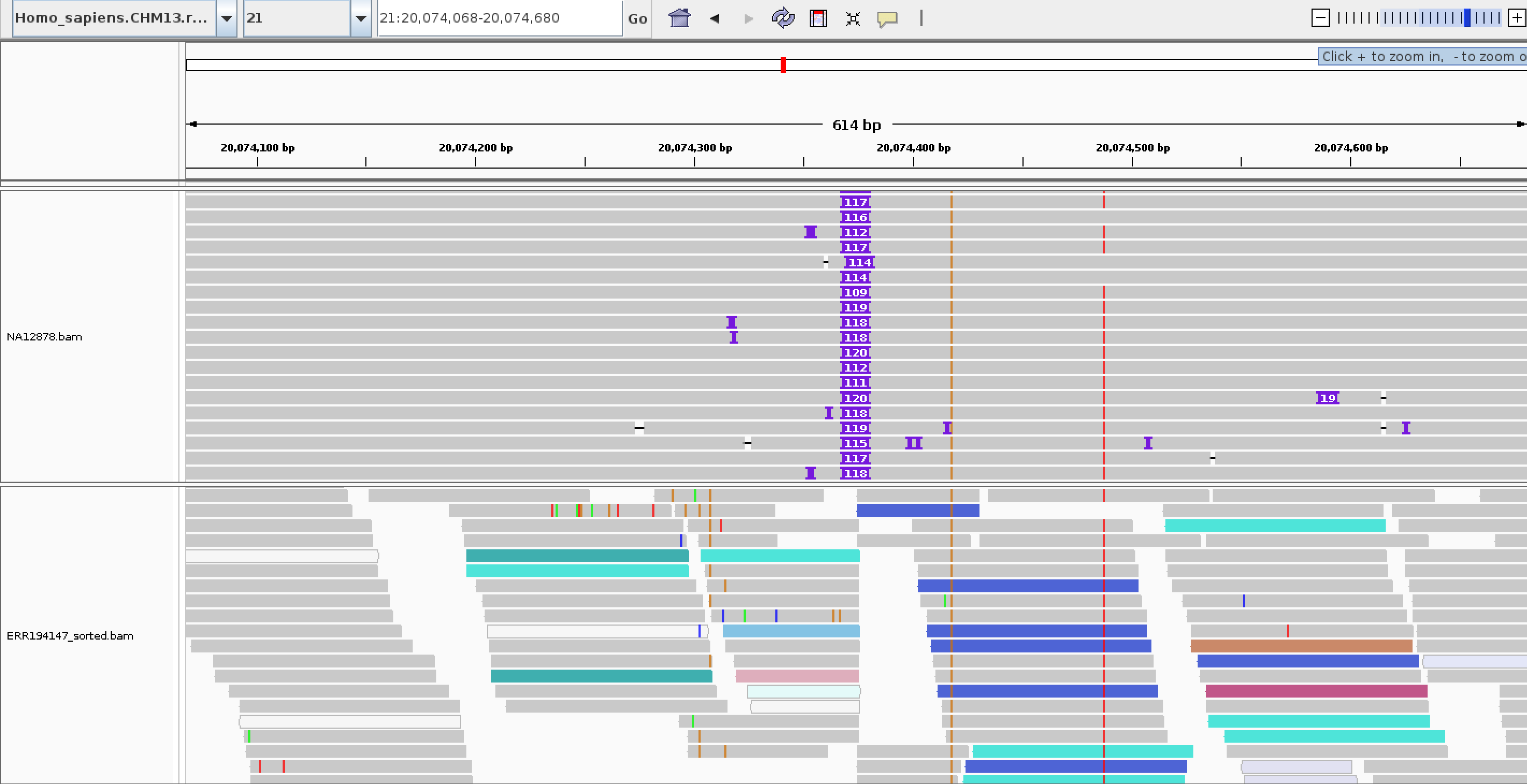
**

**Supplementary Figure 4(P):** IGV Screenshot of an NuMT called by all three methods. The NuMT is shown as an insertion in Long-read sequencing data and as discordant reads mapping to mitochondrial genome (turquoise colour) in Short-read sequencing data.

**
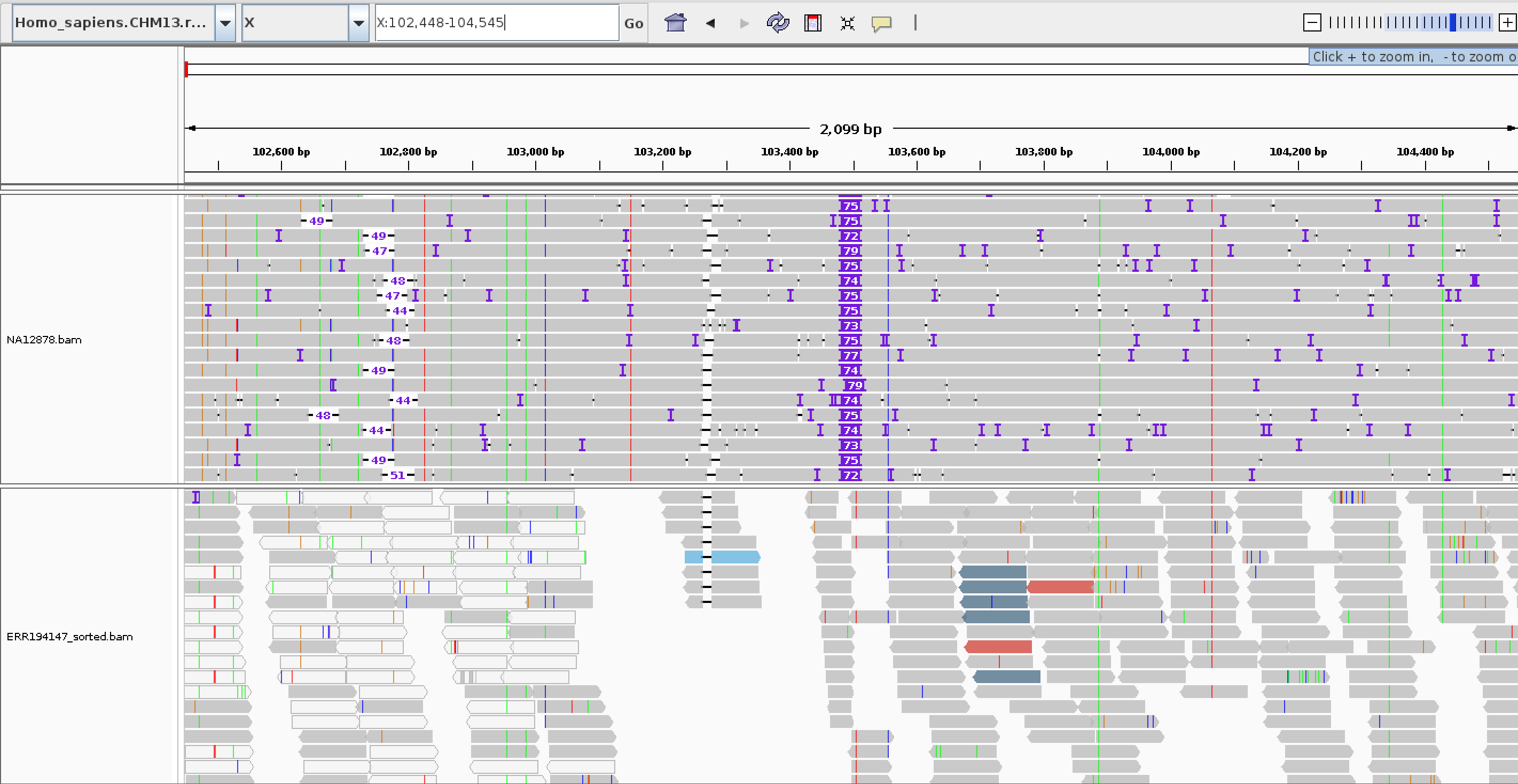
**

**Supplementary Figure 4(Q):** IGV Screenshot of an NuMT called by ANOMALY only. The NuMT is shown as an insertion in Long-read sequencing data. This NuMT is completely missed by DINUMT and Wei et al. Method.

**
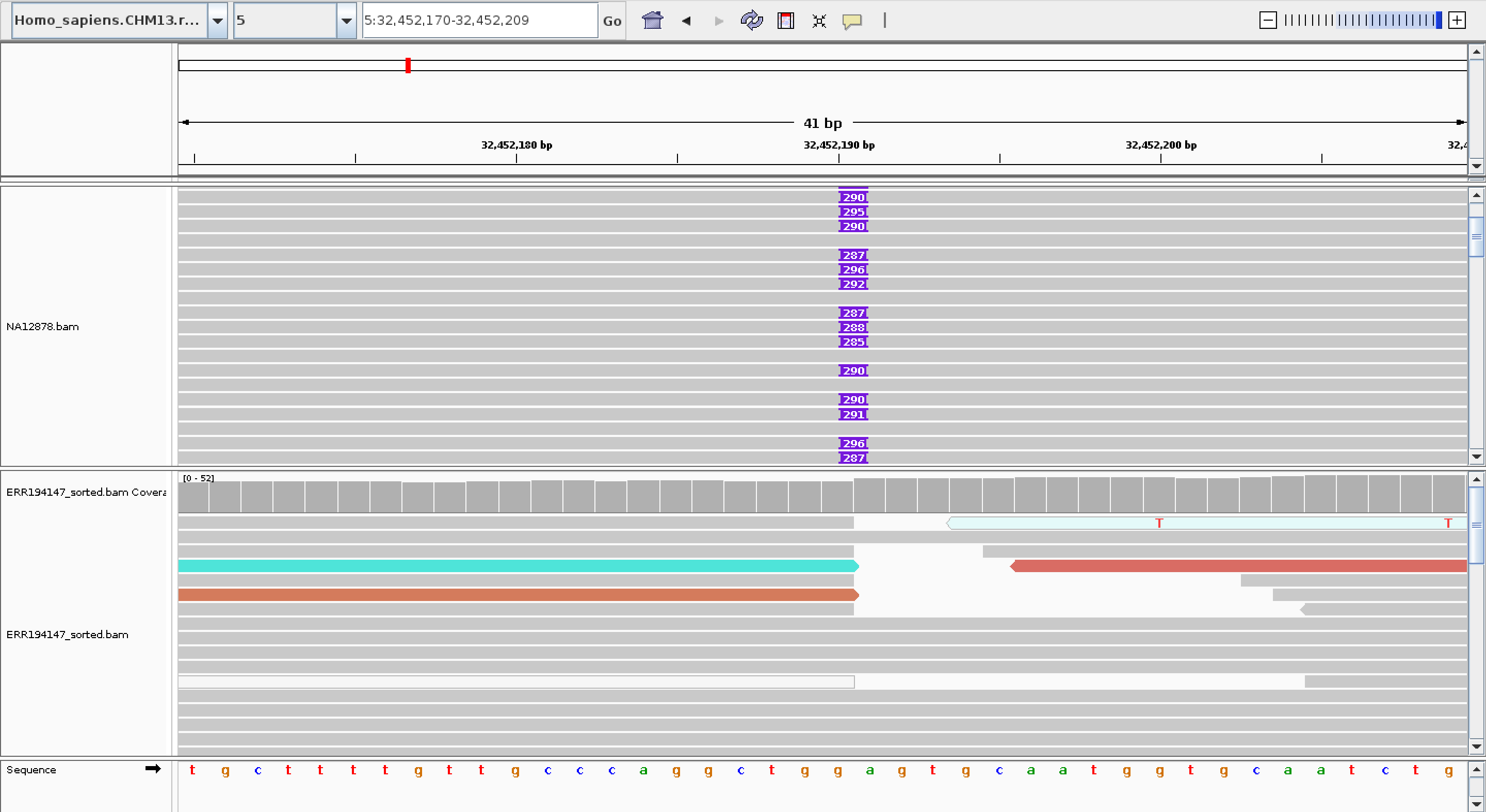
**

**Supplementary Figure 5:** IGV Screenshot showing the NuMT insertion in the long-read sequencing data of the NA12878 cell line. The NuMT shows a discrepancy when called using short-read data, as its size increases from 290 bp to 2,254 bp. Similar NuMT case was also seen in NA12889, NA12890, NA12891 and NA12892.


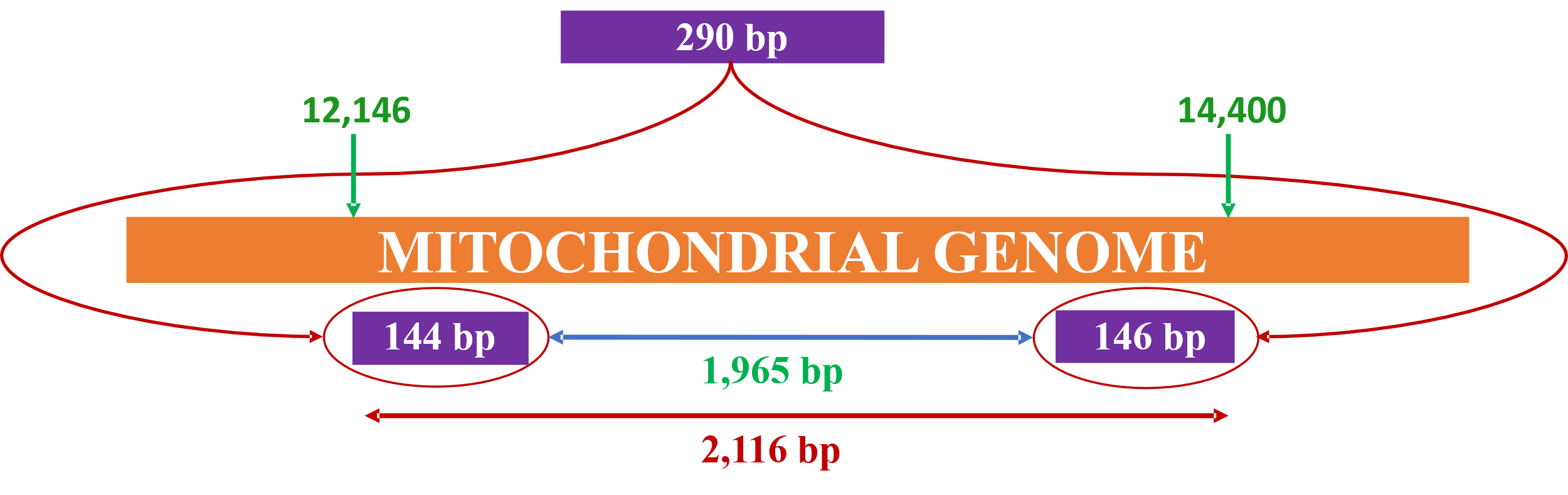


**Supplementary Figure 6:** Schematic Representation of a unique NuMT found in (NA12878, NA12889, NA12890, NA12891 and NA12892) that shows discrepancy when called using short-read sequencing data and long-read sequencing data. The short-read-based callers identified the NuMT with the size of 2,116 bp; however, the actual size is 290 bp, as identified using the long-read data. This might be because the NuMT is mapping to two parts of the mitochondrial genome that are 1,965 bp apart. Since short-read-based callers utilise discordant reads to identify the NUMTs, some reads might be mapped to the first part of the mitochondrial genome, and others might be mapped to the second part. Discordant reads mapping to two parts of the genome might be the main cause of the short-read-based callers' overestimation of NuMT size.
