# Supplementary material for: ANOMALY: a Snakemake pipeline for identifying NuMTs from long-read sequencing data
Source: NAR Genom Bioinform. 2026 Feb 4;8(1):lqag014. doi: 10.1093/nargab/lqag014 (PMC12869244; doi:10.1093/nargab/lqag014)
Supplement: lqag014_Supplemental_Files [file lqag014_supplemental_files.zip › SUPPLEMENTARY TABLES CAPTIONS.docx]

**SUPPLEMENTARY TABLES CAPTIONS**

Supplementary Table 1.xlsx contains the information regarding the tool's classification performance on simulated datasets.

Supplementary Table 2.xlsx contains the information regarding the tool's parameter optimisation to maximise the classification performance

Supplementary Table 3.xlsx contains the information regarding the tool's performance based on different SV callers

Supplementary Table 4.xlsx contains the information regarding the tool's performance based on the two branches

Supplementary Table 5.xlsx contains the information regarding the benchmark of ANOMALY with other tools on simulated datasets.

Supplementary Table 6.xlsx contains the information regarding the NuMTs missed by the tool.

Supplementary Table 7.xlsx contains the information regarding the performance of tool with respect to the sequencing depth.

Supplementary Table 8.xlsx contains the information regarding the tool's performance on NuMTs encoded by different haplogroups.

Supplementary Table 9.xlsx contains the information about the performance of the tool with respect to other methods on real-world datasets.

Supplementary Table 10.xlsx contains the information regarding the NuMTs captured by all tested methods in real world datasets.
